# An Image-Based Model of the Whole Human Heart with Detailed Anatomical Structure and Fiber Orientation

**DOI:** 10.1155/2012/891070

**Published:** 2012-08-17

**Authors:** Dongdong Deng, Peifeng Jiao, Xuesong Ye, Ling Xia

**Affiliations:** ^1^Key Lab of Biomedical Engineering of Ministry of Education, Department of Biomedical Engineering, Zhejiang University, Hangzhou 310027, China; ^2^Institute of Clinical Anatomy, Southern Medical University, Guangzhou 510515, China

## Abstract

Many heart anatomy models have been developed to study the electrophysiological properties of the human heart. However, none of them includes the geometry of the whole human heart. In this study, an anatomically detailed mathematical model of the human heart was firstly reconstructed from the computed tomography images. In the reconstructed model, the atria consisted of atrial muscles, sinoatrial node, crista terminalis, pectinate muscles, Bachmann's bundle, intercaval bundles, and limbus of the fossa ovalis. The atrioventricular junction included the atrioventricular node and atrioventricular ring, and the ventricles had ventricular muscles, His bundle, bundle branches, and Purkinje network. The epicardial and endocardial myofiber orientations of the ventricles and one layer of atrial myofiber orientation were then measured. They were calculated using linear interpolation technique and minimum distance algorithm, respectively. To the best of our knowledge, this is the first anatomically-detailed human heart model with corresponding experimentally measured fibers orientation. In addition, the whole heart excitation propagation was simulated using a monodomain model. The simulated normal activation sequence agreed well with the published experimental findings.

## 1. Introduction

Heart modeling can quantitatively study the physiological and pathological mechanism of the heart diseases, such as arrhythmias, atrial, and ventricular fibrillation, and hence to help improve their diagnosis and treatment. These developed models can also be used for medical teaching [1]. In the last several decades, a lot of research has been done on the heart modeling, from genes to the whole organ [2–4]. The hearts used for modeling were mainly from canines [5, 6], rabbits [7–9], mice [10], pigs [11], or humans [3, 12–17].

Mathematical modeling of the heart anatomy is a prerequisite for cardiac electro-mechanical simulations. Simulating the main cardiac features, including cardiac rhythms [18], mechanics [19–21], hemodynamics [22], fluid-structure interaction, energy metabolism [23], and neural control [24], can only be achieved with detailed heart structure information. It needs to be emphasized that these properties are interrelated so that any changes in one property may influence others, which makes the virtual heart modeling complicated. Therefore, giving full consideration to the anatomical structure of the heart is essential. 

 Several mathematical human heart models have been constructed to study its electrophysiological properties using the computed tomography and other modern medical imaging methods [25, 26]. However, the space resolution of these computed tomography-based heart models was not very high [12, 27]. It was 1 mm for the model developed by Lorange and Gulrajani, and the final model contained approximately 250, 000 points. The model developed by Weixue et al. [27] contained approximately 65,000 myocardial discrete units with a spatial resolution of 1.5 mm. Furthermore, neither of the two models included the conduction system. Later, human atrial models were constructed from the MRI images [16], and some of them included the atrial conduction system [14]. A human ventricular model with fiber orientations and laminar structure was constructed by Rohmer et al. [17] using the DT-MRI. In addition, the Visible Human Project provides a useful data source for detailed human heart anatomical modeling [15]. However, none of the above-mentioned models described the complete geometry of the human heart, including both the atria and the ventricles, the conduction system, and the fiber orientation. 

The conduction system plays an important role in the electrical propagation. It contains SAN, interatrial pathways, AVN, and intraventricular conduction pathways. Conduction system abnormalities could lead to cardiac arrhythmias. However, it is practically difficult to distinguish the conduction system from the surrounding tissues based on the current computed tomographic or MRI images. The heart models in early days mainly focused on the geometry of the heart without considering its conduction system [28–30]. Recently, researchers have attempted to construct the atrial conduction system [14, 31] as well as the ventricular conduction system with the His-Purkinje system [32]. However, none of the previously published models contain both the atrial and ventricular conduction systems. 

Myofiber orientation also plays an important role in the electrical conduction and mechanical contraction. Many experimental procedures have been developed to measure myofiber orientation. In early days, the measurement was usually restricted into small areas. After full thickness blocks were removed from different sites of the heart, they were cut into serial slices from epicardium to endocardium. The fiber orientation was measured from each slice [33–36]. In the early 90s, a quantitative method was developed to measure the whole ventricular fiber orientations [5], which has been widely used by other studies [6, 7, 11]. However, this method is still very timeconsuming. Advanced imaging technique, including the automated confocal microscopy, polarization microscopy and two-photon tissue cytometry, and DT-MRI, makes the measurement of fiber orientation and laminar structure possible [37–39]. However, most of these methods have been only applied to the measurement on the ventricle, not the atria.

In order to validate the function of the anatomic model, the cardiac action potential (AP) and the simulation of cardiac electrophysiology should be simulated and validated with experimental data. Until now, different AP models have been developed from different species. They were mainly based on the Hodgkin-Huxley (HH) equation, which could be used to calculate the flow of ions in the membrane and hence calculate the membrane potential changes. There are also a lot of human AP models, which include atrial working muscles [40–44], SAN [31, 45], Purkinje fibers [46, 47], and ventricle [48–51]. In our simulation, because the newly developed models are much more time-consuming and less robust in the three-dimensional simulation, the commonly used CRN model [41] and the ten Tusscher model [49] were applied to the atrial and ventricular cells. 

Regarding the simulation of cardiac electrophysiology, the reaction-diffusion equations were commonly used in combination with the anatomic and AP models. Modeling of the electrical conduction in early days was usually based on the cellular automata [2, 3, 27, 52, 53]. Later, with the enhancement of computation capacity, ionic models have been gradually applied to small-scale simulation of excitation conduction. In 1978, Tung introduced the “bidomain model” to simulate the propagation of excitation [54], However, the bidomain model requires a major dimension of the matrix inversion, and very large computing capacity. Therefore, the monodomain model was often used [55–59] because only the changes of cell membrane potential are calculated. Studies have also shown that there were no obvious differences for the computed excitation sequence between the bidomain model and monodomain model [60]. 

The aim of this study is to construct a whole human heart model with detailed anatomical structure which contains atrial and ventricular conduction systems and fiber orientations. Based on the constructed heart model, the human AP models will be assigned to different parts of the heart. Finally, the normal electrical propagation will be simulated and compared with the experimental data.

## 2. Materials and Methods

### 2.1. Cardiome-CN Human Heart Anatomical Model

#### 2.1.1. Data Acquisition

**Figure 1:**
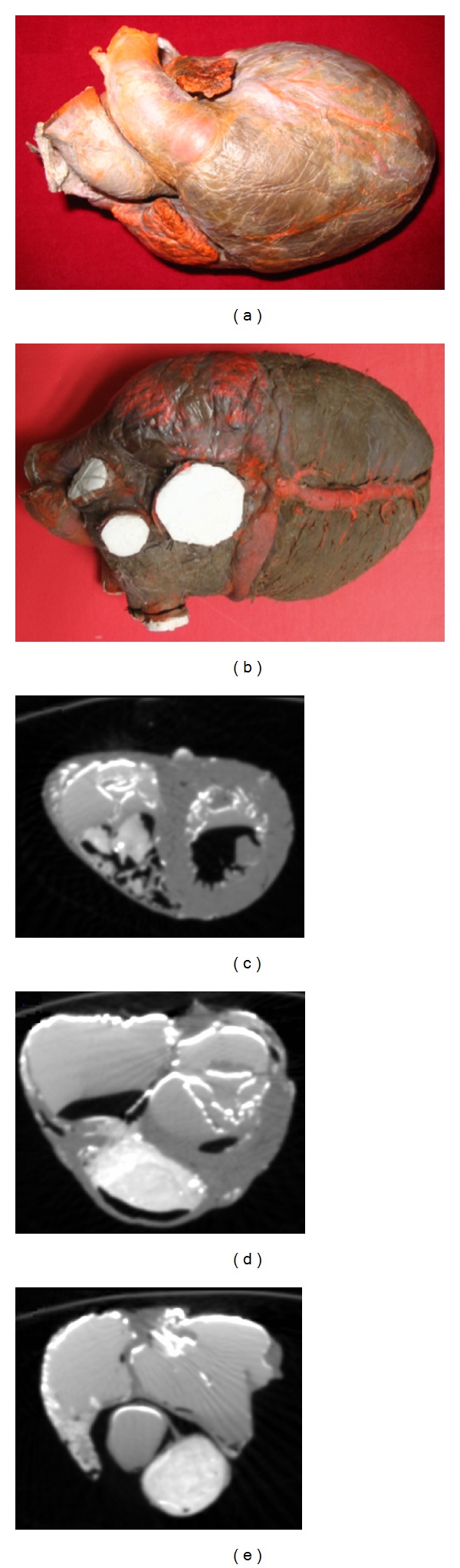
Specimen of human heart and its computed tomography (CT) images. Upper row: frontal view (a) and back view (b) of the original human heart pictures. Bottom row: three CT images from different layers of the heart, including (c) ventricle, (d) both atria and ventricle, and (e) atria.

After the confirmation of brain death, the heart specimen (Figures 1(a), 1(b)) from a healthy male adult with a tragic accident was donated to Zhujiang Hospital, Southern Medical University, P.R. China, by his family members. They gave the written consent. The use of the heart for research purpose was approved by the Ethics Committee of the Southern Medical University. The National Rules and Regulations on Heart research were strictly followed. The pretreatment of the heart and image data collection were performed at the Southern Medical University, and the follow-up works including image processing, the three-dimensional (3D) heart anatomical reconstruction was completed at Zhejiang University. 

 Firstly, the pericardium was carefully stripped off and removed with the large blood vessels (aorta and pulmonary arteries) retained for perfusion. The lead oxide and gelatin solution (gelatin and water ratio was 5%; lead oxide solution and water ratio was 25%) were then injected into the chambers of the heart through the aorta [61]. After the solution was cooled down, the heart specimen was scanned using a spiral CT (Philips/Brilliance 64). The raw CT images had a resolution of 512 pixels by 512 pixels, and the total number of images was 531 with the spatial resolution of 0.3574 mm × 0.3574 mm × 0.33 mm (Figure 1).

#### 2.1.2. Image Processing Procedure for the Construction of Human Heart Model

The commercial software ScanIP (Simpleware Inc.) was used to segment and reconstruct the anatomy of the human heart with some manual intervention to achieve the maximum accuracy. The processing procedure is briefly summarized as follows.

**Figure 2:**
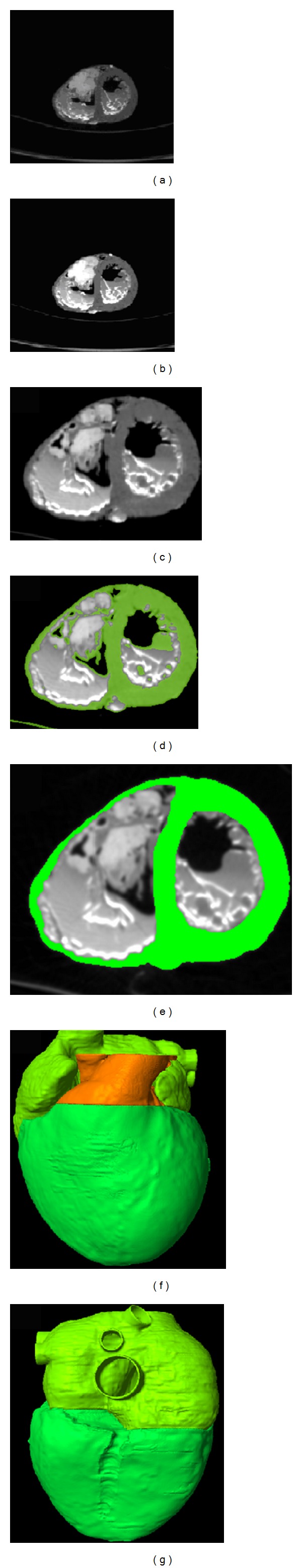
Image processing of the construction of human heart model: (a) the original CT image; (b) after contrast adjustment; (c) after cropping; (d) after contour extraction using threshold segmentation; (e) after segmentation; (f) frontal view of the reconstructed model; (g) back view of the reconstructed model.

1. Contrast adjustment: as can be seen in Figure 2(a), the contrast between myocardium and gelatin inside the heart cavity was not obvious in the original CT image. After the contrast adjustment, they could be distinguished obviously (Figure 2(b)).
2. Image cropping: since a large portion of the original CT image was from the background, they were cropped to define the area of interest and to reduce the image size to 233 × 301 (Figure 2(c)). After image cropping, the required computer memory was reduced, and the reconstruction speed was improved.
3. Contour extraction: the threshold segmentation was firstly applied to the cropped images to obtain the myocardium (Figure 2(d)). Unfortunately, some nonmyocardial tissues were wrongly included. To overcome this limitation, manual check was performed to exclude these nonmyocardial tissues. The connective tissues linked with the endocardium, such as mastoid muscle, trabecula, and so forth, were also excluded. A regional growing method was then applied to generate a clear myocardial image, as shown in Figure 2(e).
4. Image reconstruction: the above image processing procedure was repeated to all the CT images to reconstruct the heart with a surface mesh (Figures 2(f) and 2(g)).

### 2.2. Construction of Conduction System of the Cardiome-CN Human Heart Model

Mimics software (Materialise Inc) was used to construct the cardiac conduction system based on prior knowledge of the human heart anatomy. The conduction system in our model contained sinoatrial node (SAN), crista terminalis, pectinate muscles (PM), Bachmann's bundle (BB), intercaval bundles, atrioventricular node (AVN), atrioventricular ring (AVR), His bundle, bundle branches, and Purkinje network. As an example, the detailed process to construct crista terminalis is summarized as follows.

**Figure 3:**
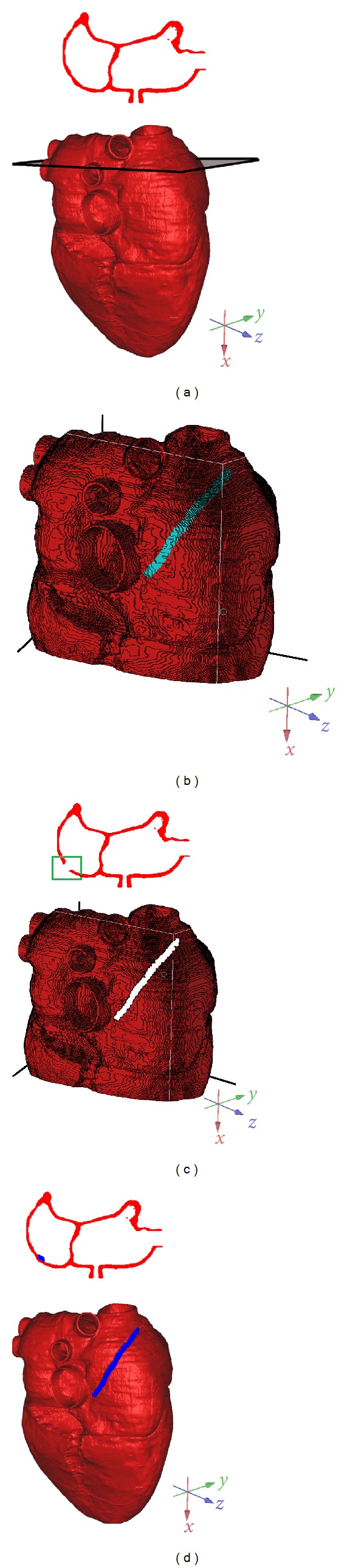
Construction of the cardiac conduction system. (a) Reconstructed 3D heart model and one cross-sectional slice of the 3D model; (b) selection of the voxels of crista terminalis in the 3D model; (c) after deletion of the voxels of crista terminalis and the corresponding 2D image; (d) final 3D heart model with one cross-sectional slice; the blue pixels show the conduction bundle.

1. Reconstruct 3D heart model from the segmented CT images: after this step, the manipulation of the voxels in the 3D model directly reflected the corresponding pixels in 2D images. However, at this stage, the conduction system was not distinguished from the heart wall, as shown in Figure 3(a).
2. Edit the CT images interactively: to separate the conduction system, the reconstructed model was depicted based on prior knowledge of the anatomical structure of the human heart. The heart model was extended from the anteromedial wall on the right side of the entrance of the superior caval vein to the right side of the entrance of the inferior caval vein [62, 63]. The voxels from the right side of the superior caval vein and downwards to the right side of the inferior caval vein were then selected as shown in Figure 3(b).
3. Project to 2D image: after the voxels of crista terminalis were obtained, they were deleted from the 3D model (the white banding in Figure 3(c)). The corresponding 2D image pixels were also deleted as marked with the green rectangle in Figure 3(c).
4. Edit the 2D images: an image erosion operation was performed to locate crista terminalis under the epicardium, and a dilation operation was used to spread crista terminalis out of the endocardium. The final 3D model was obtained as shown in Figure 3(d). The blue pixels in the upper image of Figure 3(d) show a cross-sectional image of the crista terminalis.
5. Classification and visualization: the other conduction bundles were also obtained similarly by repeating the above 4 steps.

### 2.3. Construction of Fiber Orientation of the Cardiome-CN Human Heart Model

#### 2.3.1. Fiber Orientation Acquisition

**Figure 4:**
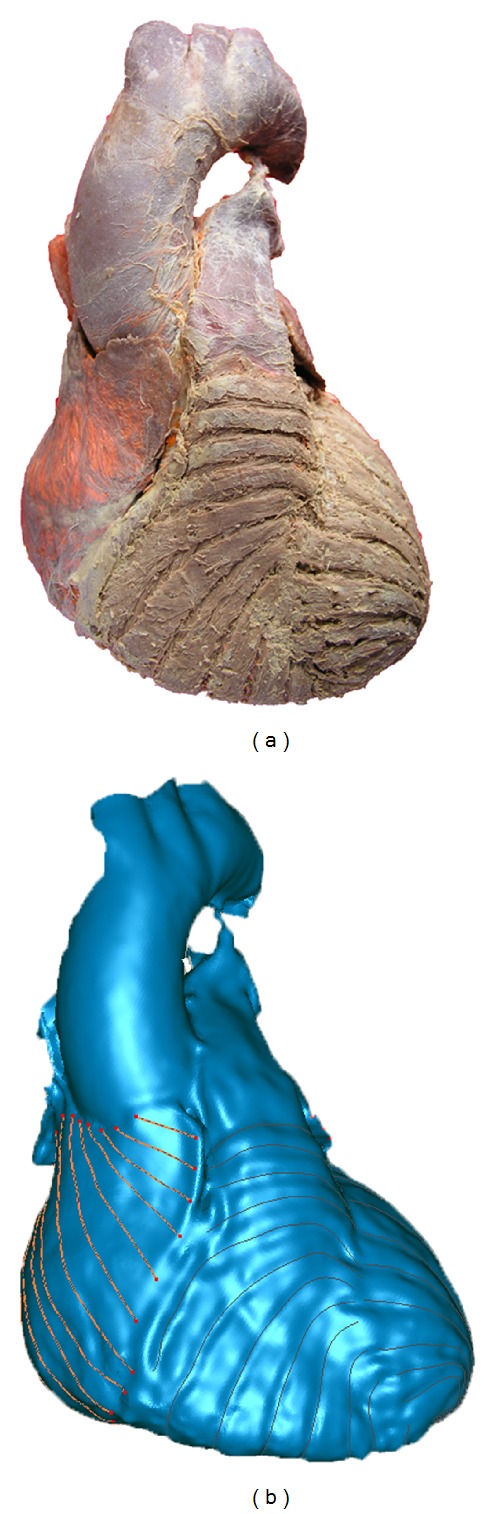
Fiber orientation acquisition. (a) The heart before laser scanning. Some atrial muscles were peeled off to make the fiber orientation visible. (b) The heart after laser scanning. The fiber orientation is represented by the red curves.

In order to obtain the fiber orientation, some atrial muscles were peeled off along the fiber orientation after the CT scan (Figure 4(a)). The heart was then scanned by a 3D laser scanner (RealScan USB Scanner model 200) with a spatial resolution of 0.01 mm. Geomagic software (Geomagic, Inc) was used to trace the fiber orientation [64]. As shown in Figure 4(b), different curves represent the fiber orientation with its coordinate information. A registration method was then applied to obtain three layers of fiber orientation at the same coordinate. The details of this method has been described in our previous publication [65].

#### 2.3.2. Construction of Ventricular Fiber Orientation

**Figure 5:**
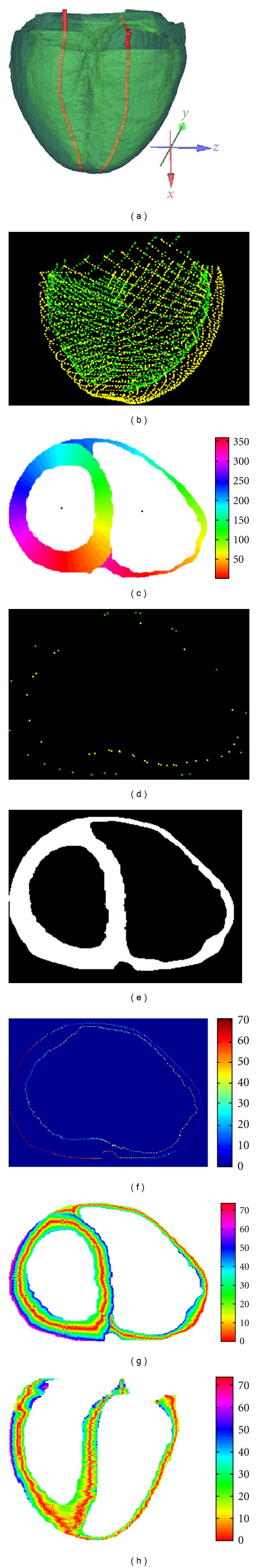
(a) Identified center of gravity of the left and right ventricles; (b) all the measured points with fiber orientation data; (c) angel of each point on a single layer; (d), (e) sorting the fiber data and the CT data of the corresponding layer; (f) fiber orientation of all the points on the epicardium and endocardium; (g) fiber orientation of the myocardial wall between epicardium and endocardium; (h) fiber orientation in a coronal plane (*xz* plane).

After the registration, the orientations of the endocardial and epicardial fibers had the same coordinate.  Figure 5(b) shows all the measured points with the fiber orientation data. These data were used to construct the whole ventricular fiber orientation. The construction steps are summarized as follows.

1. Identify the center of gravity of the left and right ventricles (the red lines in Figure 5(a)).
2. Calculate the angle of each measured point: the center of gravity was set to be origin, with *z* axis on the right side and *y* axis on the upside.  Figure 5(c) shows the angles of all the points on a single layer, with different colors representing different angels in degrees from 0°–360°.
3. Calculate the angle of each point on the direction from the apex to the base of the heart: for each layer, the points having fiber orientation at the endocardium and epicardium are marked, respectively, with yellow and green in Figure 5(d). The angle of each point was then calculated as described in step 2.
4. Match the endocardial and epicardial fiber angles with the corresponding CT data for each layer (Figure 5(e)): the linear interpolation was then used to calculate the fiber orientation of all the points on the epicardium and endocardium for each layer, as shown in Figure 5(f).
5. Calculate the fiber orientation between the epicardium and endocardium over the myocardial wall, as shown in Figure 5(g).
6. Follow steps 2–5, the fiber angles of all the layers were obtained.  Figure 5(h) shows the fiber angles in a coronal plane (*xz* plane).

#### 2.3.3. Construction of Atrial Fiber Orientation

 Due to the complexity of atrial myoarchitecture, its fiber orientation has not been well quantified. The published qualitative studies concluded that right atrial fiber orientation is obliquely aligned and has different regularity at different layers.

 In this study, only the epicardial myofiber orientation of the atria was measured. With the measured atrial fiber orientation data, the interpolation technique was applied to the whole atrial points. The construction steps are summarized as follows.

1. Calculate the inclination and transverse angles of each point with measured fiber orientation in the atria.
2. For the points without measured fiber orientation, the inclination and transverse angles from their closest point having measured fiber orientation were assigned.

### 2.4. Electrophysiological Cell Models

 For the atrial SAN, the cell model developed by Chandler et al. was used [45]. For the crista terminalis, PM, BB, and atrial working muscles, the models developed by Courtemanche et al. [41] were used. The detailed description has been given in our recently published study [66]. 

Modeling of human AVN cells is difficult, partially because there is no published study and there is no physiological parameter of human AVN cells available. In this study, a modified AP model of the atria was used to represent the AVN cell model to have the conduction time in the AVN within the physiological range [41]. There are also no existing AP models of the His bundle and bundle branches. It has been reported that Purkinje cell is the principal cell in His bundle, particularly for the left bundle branch [67, 68]. Therefore, the human Purkinje cell AP model developed by [46] was used to represent the AP models of the His bundle, left and right bundle branches. For the ventricles, the cell model developed by ten Tusscher et al. was used [49, 69]. 

### 2.5. Numerical Simulation of Excitation Conduction

The monodomain equation was used to simulate the excitation conduction, which is expressed as [70]:

(1) $$\begin{array}{c} \frac{\partial V_{m}}{\partial t}=\nabla \cdot \left(D\nabla V_{m}\right)-\frac{I_{\text{ion}}+I_{\text{applied}}}{C_{m}},\\ D=\frac{G_{i}}{S_{v}C_{m}}, \end{array}$$

where *S*_*v*_ is the surface volume ratio of cells (*μ*m^−1^), *C*_*m*_ is the specific capacitance (pF), **G**_*i*_ is the bulk intracellular conductivity (mS/cm), *V*_*m*_ is the transmembrane potential (mV), *I*_applied_ is the transmembrane stimulating current density, and *I*_ion_ is the sum of all transmembrane ionic currents (pA/pF). 

In this study, the finite difference method was used to calculate (1) because of its simplicity and suitability for the parallel computation. The time step was 0.01 ms, the anisotropy of the atrial working muscles was set as 1.3 : 1 [66], the conduction system was set as 9 : 1 [31], and the ventricular working muscles were set as 2 : 1 [55, 71].

 The simulation was performed on a Dawning TC4000L server, which had symmetric multiprocessor shared memory and contained one management node and 10 computation nodes. Each computation node contained two Intel Xeon 5335 processors, 4 G memory, and 160 G hard disk. The total theoretical computing capacity was up to 184 Gflops. MPICH2 was used to implement the communication between the computational nodes [66].

## 3. Results

### 3.1. Reconstructed Anatomical Model of the Human Heart

**Figure 6:**
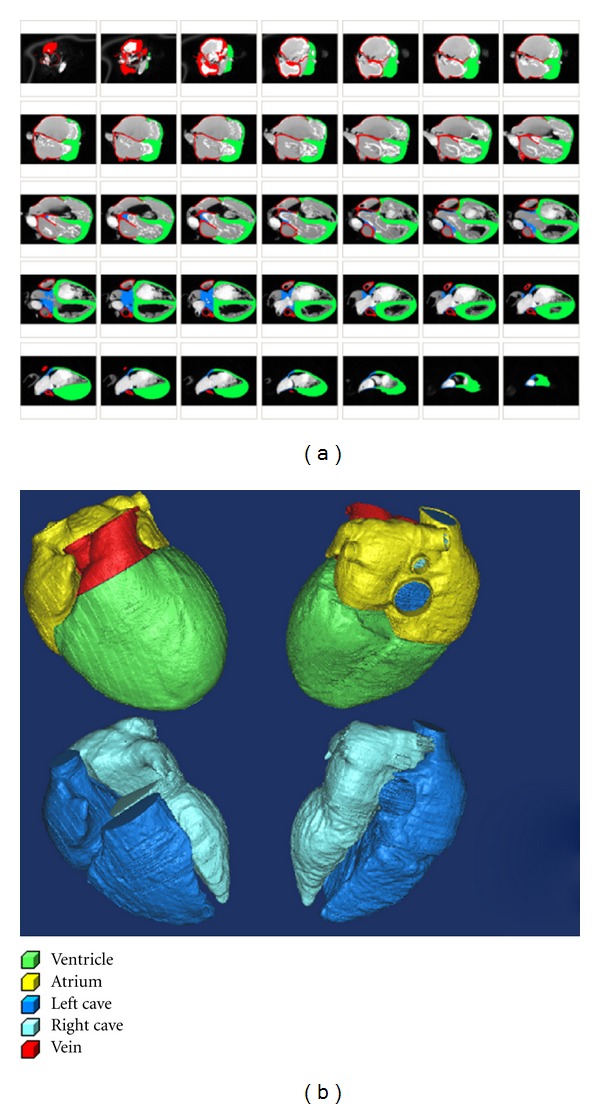
Reconstructed human heart model. (a) The segmented images. (b) The top two images show the four chambers of the heart model; the bottom two images are the inner cavity of the heart.

The reconstructed human heart anatomical model, including both the ventricles and atria, is shown in Figure 6(b). From the segmented images (Figure 6(a)), it can be seen that the left ventricular wall is much thicker than that of the right ventricle (average value: 8–10 mm versus 2–4 mm), and different layers of the ventricle have different thicknesses. The wall thickness of the atria is slightly thinner than the right ventricle. 

### 3.2. Reconstructed Conduction System of the Cardiome-CN Heart Model

The final conduction system contained the following.

**Figure 7:**
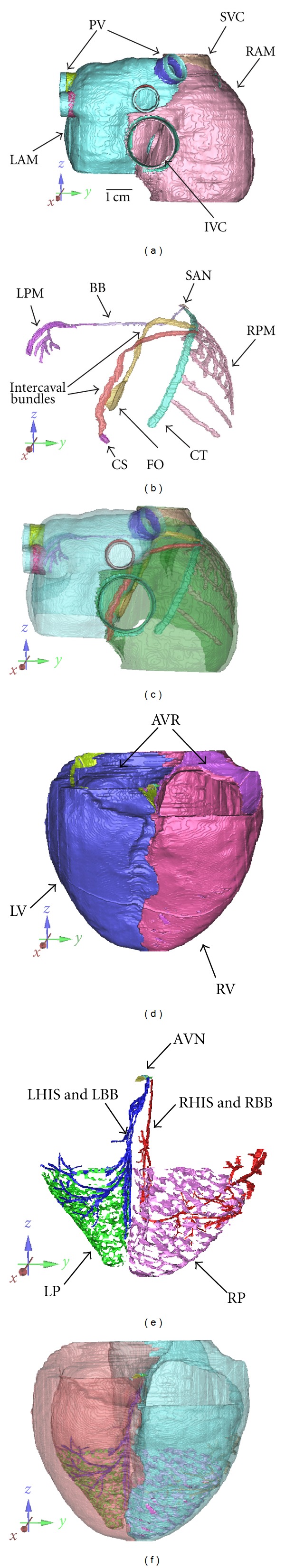
The Cardiome-CN human heart conduction system. (a) The posterior view of the atria; (b) the conduction bundles in the atria; (c) the transparent display of the conduction bundles and atrial muscles; (d) the posterior view of the ventricle; (e) the conduction bundles in the ventricle; (f) the transparent display of the conduction bundles and ventricular muscles. Note: PV: pulmonary veins; LHIS: left His bundle; RHIS: right His bundle; RBB: right bundle brunches; LBB: left bundle brunches; RP: right Purkinje fiber system; LP: left Purkinje fiber system; AVR: atrioventricular ring.

1. SAN (Figure 7(b)): including the center and periphery part. It locates at the superior poster lateral wall of the right atrium with the size of about 10 mm × 4 mm × 1 mm, which matches the published experimental data [56–59].
2. AVN (Figure 7(e)): including fast conduction region, slow conduction region, and central region [57, 72–78]. The slow conduction region was similar to the inferior nodal extension [78, 79], the fast conduction region was similar to the transitional tissue, and the central region was similar to the compact node. In our model, the size of the AVN was about 7 mm × 4 mm × 1 mm.
3. Crista terminalis and PM (Figure 7(b)): There is little quantitative data about the crista terminalis and PM, in this study, they were reconstructed from the qualitative description of the position and anatomic structure [62, 63]. The crista terminalis was extended from the anteromedial wall on the left side of the entrance of the superior caval vein to the right of the entrance of the inferior caval vein. PM are parallel alignment of the muscle bulges on the appendage wall and the posterior wall of the right atria [62].
4. Intercaval bundles (Figure 7(b)): one bundle connects the origin of the crista terminalis and the anterosuperior rim of FO, and the other connects the origin of the crista terminalis and CS. The details have been reported in our previous publication [66].
5. BB (Figure 7(b)): the length of the BB is 14.7 mm, and the maximum diameters of the anteroposterior and superoinferior are 4.5 mm and 3.7 mm [62, 80–82].
6. His bundle, left and right bundle brunches, and Purkinje fiber system (Figure 7(e)): they were constructed from the published data [68, 83–88]. Left bundle branch starts from the bifurcation of the atrioventricular bundle, descends along the interventricular septum about 1.5 cm, and is then divided into three branches. The right bundle branch starts from the end of the bifurcation of the atrioventricular bundle, moves downward along the membranous part of interventricular septum, and passes the papillary muscle of the conus to the moderator band. After reaching the root of prepapillary muscles, it is divided into three branches. The Purkinje fibers reach into the ventricular myocardial to form the subendocardial network. It is mainly located in the lower part of the interventricular septum, apex, papillary muscles, and free wall. In our model, the Purkinje fiber system, not the His bundle and left and right bundle brunches, conducts the excitation to the surrounding ventricular working muscles.

### 3.3. Reconstructed Fiber Orientation of the Cardiome-CN Heart Model

**Figure 8:**
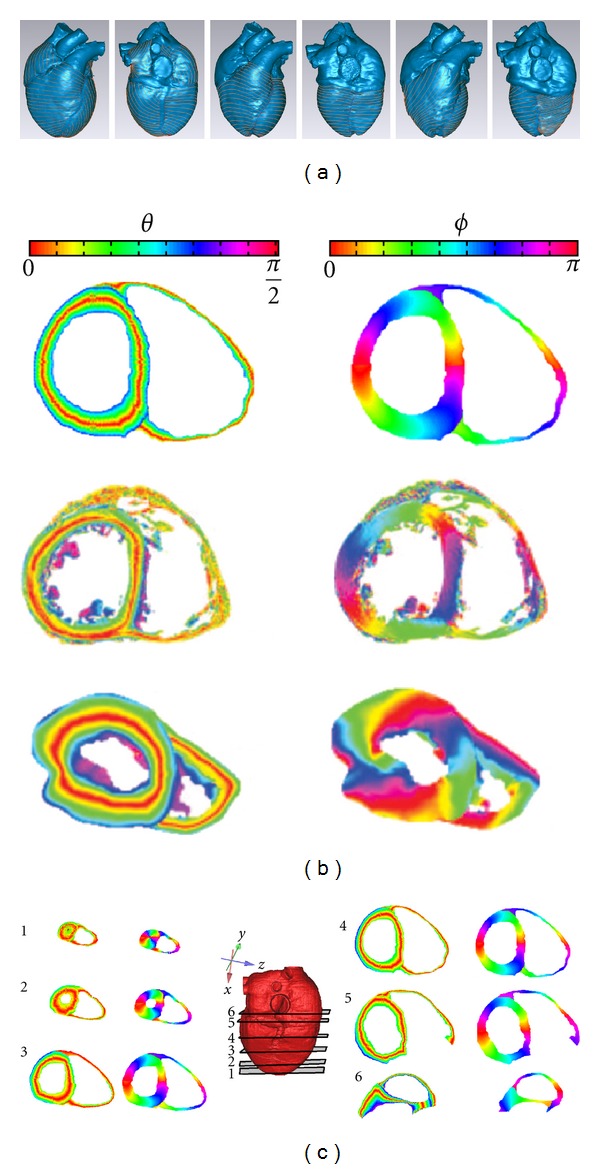
(a) Original fiber direction with frontal and back view: the two images from the left are the epicardial fiber orientation; the middle two images are the middle layer fiber orientation; the two images from the right are the subendocardial fiber orientation. (b) Ventricular fiber orientation, with *θ* the inclination angle and *ϕ* the transverse angle. The first row is the constructed fiber orientation of our model, the second row is the published human ventricular DTMRI data, and the third row was the data extracted from [55]. (c) Constructed fiber orientation of the cross sections of the ventricle. The numbers represent the position of cross sections in the whole heart model.

On the ventricular epicardium (see the first two images from the left in Figure 8(a)), fibers start from the atrioventricular junction and extend obliquely to the cardiac apex along the blunt edge. Near the atrioventricular junction area, longitudinally oriented fibers are observed. When crossing the blunt edge and close to the posterior sulcus, the fiber orientation is transverse. The fiber orientation of the diaphragmatic surface of the right ventricle is nearly circumferential until it crosses the sharp edge. When close to the outlet of the right ventricle, it is perpendicular to the plane. The fiber orientation in the anterior interventricular groove does not continue, but it forms an angle.

On the middle layer of the anterior and on the posterior and lateral walls of the left ventricle, the fibers are nearly circumferential (see the middle two images in Figure 8(a)), and the fiber in the diaphragmatic surface of the right ventricle middle layer is also circumferential. But when crossing the blunt edge, the fiber orientation is a little oblique, then changes to circumferential again in the anterior wall. When close to the outlet of the right ventricle, the fiber becomes steep and the orientation is longitudinal. Different with the ventricular epicardial junctional area, the fiber continues on the middle layer junctional area of the right and left ventricles.

 On the endocardium (see the two images from the right in Figure 8(a)), the fiber orientation is more oblique on the anterior wall than the posterior wall. Overall, from the apex to the base of the heart, the epicardial fibers are arranged clockwise, and the endocardial fibers are counterclockwise. From the epicardium to endocardium, the fiber orientation changes continuously, but inconsistently at different parts.

Figure 8(b) gives the comparison between our results and these from other groups. The first row in Figure 8(b) is one cross-section of the fiber orientation from our model, the second row is the published human ventricular DTMRI data [8, 55, 89], and the third row is the data extracted from [55]. It clearly shows that our result was very similar with the DIMRI data.  Figure 8(c) shows the constructed fiber orientation of the cross-sections of the ventricle, with the inclination and transverse angles given.

**Figure 9:**
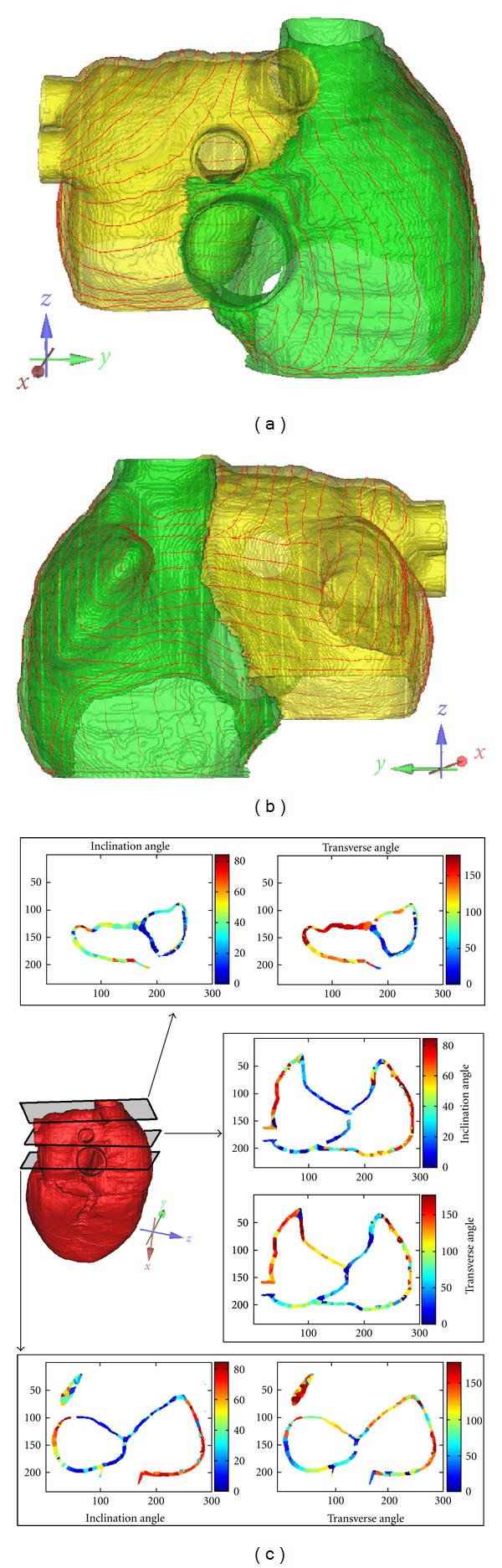
Constructed atria fiber orientations. (a), (b) Anterior view and posterior view of the fiber orientation within the atrial anatomical model; (c) the constructed fiber orientation of three cross-sectional images of the atria model. The fiber orientation in each layer is represented by inclination and transverse angles.

Figure 9 shows the atrial anatomical model with the fiber orientation. The atrial fiber orientation is much more complex than the ventricle. On the posterior and lateral wall of the right atrium, the main fiber direction is longitudinally aligned. The fibers begin in the junction area of the superior vena cava and extend to the atrioventricular junction. Because of the pulmonary veins in the left atrium, the fiber orientation is not as regular as the right atrium. On the posterior and upper posterior wall of the left atrium, fiber orientation is inclined, with a greater inclination on the upper posterior wall. On the lateral wall of left atrium from the left superior pulmonary vein to the apex of the left auricular appendage, it is also inclined. At the top of the left atrium, it is inclinable from the left and right pulmonary veins to the left atrial appendage and interatrial septum, respectively, and is fused on the anterior wall of the atrium. Figures 9(a) and 9(b) are the atrial anatomic model with the fiber orientation, and Figure 9(c) is the final atrial fiber orientation of some selected layers of the atrial model; each layer is represented by inclination and transverse angles.

### 3.4. Simulation Results of the Cardiac Electrical Propagation

**Figure 10:**
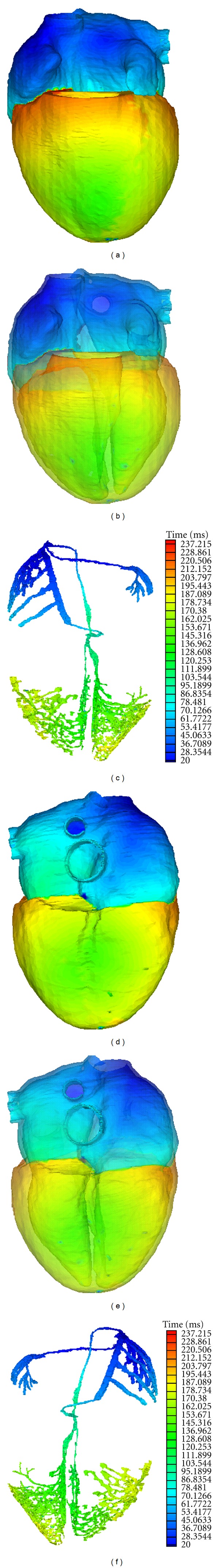
The simulated excitation sequences of the whole human heart. Different colors represent different excitement time. (a, d) the front and back views of the excitation sequence; (b, e) the transparence view; (c, f) the excitation sequence of the conduction systems.

The excitation sequence of the human heart is shown in Figure 10. The frequency of the pacemaker in our model was 1.19 Hz. The excitation starts from the SAN, reaches the BB and crista terminalis in right atrium after approximately 10 ms, and conducts to AV junction via the FP, SP, and crista terminalis. It then quickly conducts to the APG. After about 50 ms the left atrial septum close to the fossa ovalis is activated. 

The current originated in the SAN also conducts excitation to the left atrium via the BB. In the left atrium, the excitation initiates from the region near the BB, then conducts to the APG via the right PM and to the posterior part of the right atrium, and ends at the posterior-inferior left atrium. The complete activation time is 30 ms for BB, 81 ms for the right atrium, 79 ms for left atrium, and 109 ms for the entire atrium. The conduction velocity is about 115 cm/s, 76 cm/s, 125 cm/s, and 107 cm/s in the crista terminalis, atrial muscles, PM, and BB, respectively. 

When the excitation conducts to the AVN, with a delay of about 15 ms, it reaches the His bundle, left and right bundle, and then to the Purkinje network. The first breakthrough in the endocardium is at about 136 ms in the lower septum, and then the excitation conducts to the ventricular cells via Purkinje network. The first breakthrough point in the epicardium is at the anterior and posterior septal region, which is consistent with clinical measurements [90]. In the left and right ventricles, the last activated regions are the posterolateral area and the pulmonary conus and posterobasal area. It happens at about 228 ms. The conduction velocity varies at different parts, with the slowest speed of 40 cm/s at the apex, about 70 cm/s at the middle part of the heart and, about 80 cm/s at the upper part close to the base of the ventricle.

## 4. Discussion

### 4.1. Fiber Orientation Modeling

 The fiber orientations of the ventricles and atria have been investigated in this study. The human ventricular fiber orientation has been widely studied with the range of +60° ~ −60°, depending on the different species used [5, 8, 91–93]. Our results agreed well with what has been published [17, 36, 94, 95]. Moreover, our results quantitatively showed that the fiber orientation is not homogeneous on the same layer and also varies at different parts of the heart. It is more inclined near the orifice of pulmonary trunk than the middle and bottom parts of the ventricles, and on the diaphragmatic surface of the epicardium, the left ventricular fiber has steeper angles than the right ventricle. 

 In this present study, the epicardial fiber orientation of the atrium was quantitatively measured, and the result shows that the atrial fiber orientation in general is less regular than that of the ventricle. On the right atrial epicardium, the fiber has some patterns, but in the left atrium, it is quite irregular because of the pulmonary veins. Our data was consistent with other experimental data [62, 96, 97]. 

### 4.2. Conduction System Modeling

A detailed cardiac conduction system has been constructed in this study. It is very difficult to distinguish different conduction pathways using the anatomic or morphological methods. In our model, it has been assumed that muscle bundles exist between the origin of crista terminalis and CS and FO, and they compose of normal atrial muscles, but have high anisotropy ratio. The geometry of the two pathways agreed the general description of the experimental data [96–98]. To the best of our knowledge, this is the first model containing the two pathways in biatrial conduction simulation. Due to the lack of accurate experimental data, the accurate geometry of the two pathways can not be obtained in our simulation, but our simulation showed that they could make the conduction pattern in the atria more close to the clinic data [66].

Recently, it has been reported that the SAN structure was functionally insulated from the atrium by the connective tissues, fat, and coronary arteries [99]. It has also been reported that the atrial myocardium was excited via the superior, middle, and/or inferior sinoatrial conduction pathways. Therefore, in our simulation the excitation from the periphery cell only conducts to the crista terminalis, and the two internodal bundles are supposed to be from near the SAN. In the other atrial simulations [14, 31], the SAN could conduct electricity to the surrounding atrial working muscles, because the SAN was considered not to be insulated from the surrounding tissues.

Due to the complexity of the AVN, its anatomy and morphology have not been fully understood. In our model, based on the theory of the dual AVN pathways [78, 100–102], the AVN is divided into three parts: fast and slow conduction regions, and central region. The fast conduction region receives the electrical excitation from the transitional cells, and the slow conduction region receives the electrical excitation from the isthmus. These settings may have some differences with the real anatomic structure, but it made the whole heart modeling become feasible.

Many models have been constructed to simulate the electrical conduction in the ventricle and they have different resolutions and the construction methods varies too, but the majority of them were artificially depicted based on the prior knowledge of the anatomical structure [103, 104] or special algorithms [105, 106]. The His-Purkinje system is important for the ventricular conduction, and there are many qualitative anatomical descriptions [84–88, 107–109]. In our model, the His-Purkinje system was artificially depicted based on the prior knowledge. The His and bundle branches are separated from the Purkinje network, and the Purkinje fibers are connected each other. Our model was also close to the recently constructed His-Purkinje system of a rabbit from macroscopic images [110]. 

### 4.3. Excitation Conduction Modeling

The simulated excitation sequences of the atria and ventricles in our model agreed well with the published experimental data [30, 111]. The conduction velocity in the right atrial wall is not homogenous; the velocity of the posterolateral wall with PM is 70 to 100 cm/s and the average velocity is close to 95 cm/s, which is within the range of 0.68–1.03 m/s [112]. The velocity of crista terminalis is 1.15 m/s, which is also consistent with the experimental data of 0.7–1.3 m/s [113]. Lemery et al. [111] reported that the total BB conduction time was 23 ± 15 ms, our result of 16 ms is slightly shorter than the average value, but is within the range. The conduction speed of BB in our simulation is between 95 to 150 cm/s, and the average velocity is 113 cm/s; it is within the range reported by Dolber and Spach [114]. 

De et al. [115] reported that the duration of left atrial propagation and whole atria was 81 ± 10 and 105 ± 9 ms, respectively. The experimental data in [111] showed the activation time of LA and whole atria were 80 ± 11 ms and 120 ± 24 ms, respectively, while the results of [116] were 84 ± 14 ms and 119 ± 14 ms, respectively. In our simulation, the total activation time of left atrial and whole atria are 79 ms and 109 ms, respectively, which is similar to these experimental data.

For the ventricle, the conduction time from the onset of His to the onset of ventricular working muscles is about 41 ms, which is within the range of 47 ± 7 ms [117] and close to another simulation result [32]. After the excitation of lower septum in endocardium, the electricity spread to the epicardium and then upward to the base of ventricle; the whole conduction time of the ventricular working muscles is about 92 ms, which is close to the experimental data of about 100 ms [30, 117] and another model study [118]. The conduction speed of ventricular muscles is about 0.7 m/s, close to 0.6 m/s [119] and fall in the range of 0.3–1.0 m/s [120].

## 5. Limitation

 Firstly, the mastoid muscles could not be clearly distinguished from the ventricular muscles. This is especially obvious in the right ventricle, therefore the mastoid muscles were not included in our heart model. Secondly, our model only contained the atrial epicardial fiber orientation, since the atrial wall is too thin and the endocardial fiber is complex. Thirdly, the AP model of the AVN was based on the human atrial model. It may not effectively represent the physiological parameters of human AVN cells and may influence the simulation accuracy. In addition, the anatomy of whole heart conduction system was constructed based on the priori knowledge of the heart anatomy, which may affect the simulation.

## 6. Conclusion

In conclusion, a human heart model with detailed anatomical structure, conduction system, and fiber orientations have been constructed. To the best of our knowledge, this is the first anatomically-detailed human heart model with corresponding experimentally measured fibers orientation. Different AP cell models have been assigned to different parts of the heart, and the simulated normal activation sequence agreed well with the published experimental data. Such detailed anatomical heart model could be very useful for future research on understating of the mechanisms influencing cardiovascular function and its physiological and pathological processes.

## References

[1] Wong KC, Wang L, Zhang H, Liu H, Shi P Integrating functional and structural images for simultaneous cardiac segmentation and deformation recovery.

[2] Weixue L, Ling X (1996). Computer simulation of epicardial potentials using a heart-torso model with realistic geometry. *IEEE Transactions on Biomedical Engineering*.

[3] Weixue L, Ling X (1995). Three-dimensional simulation of epicardial potentials using a microcomputer-based heart-torso model. *Medical Engineering and Physics*.

[4] Noble D (2002). Modeling the heart—from genes to cells to the whole organ. *Science*.

[5] Nielsen PMF, Le Grice IJ, Smaill BH, Hunter PJ (1991). Mathematical model of geometry and fibrous structure of the heart. *American Journal of Physiology—Heart and Circulatory Physiology*.

[6] LeGrice IJ, Hunter PJ, Smaill BH (1997). Laminar structure of the heart: a mathematical model. *American Journal of Physiology—Heart and Circulatory Physiology*.

[7] Vetter FJ, McCulloch AD (1998). Three-dimensional analysis of regional cardiac function: a model of rabbit ventricular anatomy. *Progress in Biophysics and Molecular Biology*.

[8] Scollan DF, Holmes A, Zhang J, Winslow RL (2000). Reconstruction of cardiac ventricular geometry and fiber orientation using magnetic resonance imaging. *Annals of Biomedical Engineering*.

[9] Burton RAB, Plank G, Schneider JE (2006). Three-dimensional models of individual cardiac histoanatomy: tools and challenges. *Annals of the New York Academy of Sciences*.

[10] Henriquez CS, Tranquillo JV, Weinstein D, Zipes DP, Jalife J (2004). Three dimensional propagation in mathematic models: integrative model of the mouse heart. *Cardiac Electrophysiology from Cell to Bedside*.

[11] Stevens C, Remme E, LeGrice I, Hunter P (2003). Ventricular mechanics in diastole: material parameter sensitivity. *Journal of Biomechanics*.

[12] Lorange M, Gulrajani RM (1993). A computer heart model incorporating anisotropic propagation. I. Model construction and simulation of normal activation. *Journal of Electrocardiology*.

[13] Hren R, Nenonen J, Horáček BM (1998). Simulated epicardial potential maps during paced activation reflect myocardial fibrous structure. *Annals of Biomedical Engineering*.

[14] Harrild D, Henriquez C (2000). A computer model of normal conduction in the human atria. *Circulation Research*.

[15] Sachse FB, Werner CD, Stenroos MH, Schulte RF, Zerfass P, Dössel O Modeling the anatomy of the human heart using the cryosection images of the Visible Female dataset.

[16] Blanc O (2002). *A computer model of human atrial arrhythmia [M.S. thesis]*.

[17] Rohmer D, Sitek A, Gullberg GT (2007). Reconstruction and visualization of fiber and laminar structure in the normal human heart from ex vivo diffusion tensor magnetic resonance imaging (DTMRI) data. *Investigative Radiology*.

[18] Colli-Franzone P, Guerri L, Taccardi B (2004). Modeling ventricular excitation: axial and orthotropic anisotropy effects on wavefronts and potentials. *Mathematical Biosciences*.

[19] Xia L, Huo M, Wei Q, Liu F, Crozier S (2005). Analysis of cardiac ventricular wall motion based on a three-dimensional electromechanical biventricular model. *Physics in Medicine and Biology*.

[20] Long Q, Merrifield R, Xu XY, Kilner P, Firmin DN, Yang GZ (2008). Subject-specific computational simulation of left ventricular flow based on magnetic resonance imaging. *Proceedings of the Institution of Mechanical Engineers H*.

[21] Dou J, Xia L, Zhang Y (2009). Mechanical analysis of congestive heart failure caused by bundle branch block based on an electromechanical canine heart model. *Physics in Medicine and Biology*.

[22] Smith NP (2004). A computational study of the interaction between coronary blood flow and myocardial mechanics. *Physiological Measurement*.

[23] Matsuoka S, Sarai N, Kuratomi S, Ono K, Noma A (2003). Role of individual ionic current systems in ventricular cells hypothesized by a model study. *Japanese Journal of Physiology*.

[24] Olufsen MS, Tran HT, Ottesen JT (2006). Modeling baroreflex regulation of heart rate during orthostatic stress. *American Journal of Physiology—Regulatory Integrative and Comparative Physiology*.

[25] Rudy Y (2000). From genome to physiome: integrative models of cardiac excitation. *Annals of Biomedical Engineering*.

[26] Hunter PJ, Crampin EJ, Nielsen PMF (2008). Bioinformatics, multiscale modeling and the IUPS Physiome Project. *Briefings in Bioinformatics*.

[27] Weixue L, Zhengyao X, Yingjie F (1993). Microcomputer-based cardiac field simulation model. *Medical and Biological Engineering and Computing*.

[28] Miller WT, Geselowitz DB (1978). Simulation studies of the electrocardiogram. I. The normal heart. *Circulation Research*.

[29] Miller WT, Geselowitz DB (1978). Simulation studies of the electrocardiogram. II. Ischemia and infarction. *Circulation Research*.

[30] Durrer D, van Dam RT, Freud GE, Janse MJ, Meijler FL, Arzbaecher RC (1970). Total excitation of the isolated human heart. *Circulation*.

[31] Seemann G, Höper C, Sachse FB, Dössel O, Holden AV, Zhang H (2006). Heterogeneous three-dimensional anatomical and electrophysiological model of human atria. *Philosophical Transactions of the Royal Society A*.

[32] Tusscher KHWJT, Panfilov AV (2008). Modelling of the ventricular conduction system. *Progress in Biophysics and Molecular Biology*.

[33] Streeter DD, Spotnitz HM, Patel DP, Ross J, Sonnenblick EH (1969). Fiber orientation in the canine left ventricle during diastole and systole. *Circulation Research*.

[34] Streeter DD, Hanna WT (1973). Engineering mechanics for successive states in canine left ventricular myocardium. II. Fiber angle and sarcomere length. *Circulation Research*.

[35] Carew TE, Covell JW (1979). Fiber orientation in hypertrophied canine left ventricle. *The American Journal of Physiology*.

[36] Greenbaum RA, Ho SY, Gibson DG (1981). Left ventricular fibre architecture in man. *British Heart Journal*.

[37] Young AA, Legrice IJ, Young MA, Smaill BH (1998). Extended confocal microscopy of myocardial laminae and collagen network. *Journal of Microscopy*.

[38] Axer H, Keyserlingk DGV (2000). Mapping of fiber orientation in human internal capsule by means of polarized light and confocal scanning laser microscopy. *Journal of Neuroscience Methods*.

[39] Sands GB, Gerneke DA, Hooks DA, Green CR, Smaill BH, Legrice IJ (2005). Automated imaging of extended tissue volumes using confocal microscopy. *Microscopy Research and Technique*.

[40] Grandi E, Pandit SV, Voigt N (2011). Human atrial action potential and Ca^2+^ model: sinus rhythm and chronic atrial fibrillation. *Circulation Research*.

[41] Courtemanche M, Ramirez RJ, Nattel S (1998). Ionic mechanisms underlying human atrial action potential properties: insights from a mathematical model. *American Journal of Physiology—Heart and Circulatory Physiology*.

[42] Nygren A, Fiset C, Firek L (1998). Mathematical model of an adult human atrial cell: the role of K^+^ currents in repolarization. *Circulation Research*.

[43] Maleckar MM, Greenstein JL, Giles WR, Trayanova NA (2009). K^+^ current changes account for the rate dependence of the action potential in the human atrial myocyte. *American Journal of Physiology—Heart and Circulatory Physiology*.

[44] Koivumäki JT, Korhonen T, Tavi P (2011). Impact of sarcoplasmic reticulum calcium release on calcium dynamics and action potential morphology in human atrial myocytes: a computational study. *PLoS Computational Biology*.

[45] Chandler NJ, Greener ID, Tellez JO (2009). Molecular architecture of the human sinus node insights into the function of the cardiac pacemaker. *Circulation*.

[46] Stewart P, Aslanidi OV, Noble D, Noble PJ, Boyett MR, Zhang H (2009). Mathematical models of the electrical action potential of Purkinje fibre cells. *Philosophical Transactions of the Royal Society A*.

[47] Sampson KJ, Iyer V, Marks AR, Kass RS (2010). A computational model of Purkinje fibre single cell electrophysiology: implications for the long QT syndrome. *Journal of Physiology*.

[48] Priebe L, Beuckelmann DJ (1998). Simulation study of cellular electric properties in heart failure. *Circulation Research*.

[49] Ten Tusscher KHWJ, Noble D, Noble PJ, Panfilov AV (2004). A model for human ventricular tissue. *American Journal of Physiology—Heart and Circulatory Physiology*.

[50] O’Hara T, Virág L, Varró A, Rudy Y (2011). Simulation of the undiseased human cardiac ventricular action potential: model formulation and experimental validation. *PLoS Computational Biology*.

[51] Fink M, Noble D, Virag L, Varro A, Giles WR (2008). Contributions of HERG K^+^ current to repolarization of the human ventricular action potential. *Progress in Biophysics and Molecular Biology*.

[52] Plonsey R, Barr RC (1987). Mathematical modeling of electrical activity of the heart. *Journal of Electrocardiology*.

[53] Chernyak YB, Feldman AB, Cohen RJ (1997). Correspondence between discrete and continuous models of excitable media: trigger waves. *Physical Review E*.

[54] Tung L (1978). *A Bi-Domain Model for descriBing Ischemic Myocardial D-C Potentials*.

[55] Ten Tusscher KHWJ, Hren R, Panfilov AV (2007). Organization of ventricular fibrillation in the human heart. *Circulation Research*.

[56] Anderson KR, Yen Ho S, Anderson RH (1979). Location and vascular supply of sinus node in human heart. *British Heart Journal*.

[57] James TN (2002). Structure and function of the sinus node, AV node and his bundle of the human heart—part I: structure. *Progress in Cardiovascular Diseases*.

[58] Dobrzynski H, Li J, Tellez J (2005). Computer three-dimensional reconstruction of the sinoatrial node. *Circulation*.

[59] Fedorov VV, Glukhov AV, Chang R (2010). Optical mapping of the isolated coronary-perfused human sinus node. *Journal of the American College of Cardiology*.

[60] Potse M, Dubé B, Richer J, Vinet A, Gulrajani RM (2006). A comparison of monodomain and bidomain reaction-diffusion models for action potential propagation in the human heart. *IEEE Transactions on Biomedical Engineering*.

[61] Lou X (2006). Modified lead oxide-gelatin injection technique for angiography. *Chinese Journal of Clinical Anatomy*.

[62] Ho SY, Sánchez-Quintana D (2009). The importance of atrial structure and fibers. *Clinical Anatomy*.

[63] Sánchez-Quintana D, Anderson RH, Cabrera JA (2002). The terminal crest: morphological features relevant to electrophysiology. *Heart*.

[64] Zhang MC, Liao JM, Li M, Zhao WD (2004). Reconstruction of the mandibular model using a three-dimensional laser scanner. *Journal of Southern Medical University*.

[65] Deng D, Jiao P, Shou G, Xia L Registering myocardial fiber orientations with heart geometry using iterative closest points algorithms.

[66] Deng D, Gong Yl, Shou G Simulation of biatrial conduction via different pathways during si-nus rhythm with a detailed human atrial model.

[67] James TN, Sherf L (1971). Fine structure of the His bundle. *Circulation*.

[68] James TN, Sherf L, Urthaler F (1974). Fine structure of the bundle branches. *British Heart Journal*.

[69] Ten Tusscher KHWJ, Panfilov AV (2006). Cell model for efficient simulation of wave propagation in human ventricular tissue under normal and pathological conditions. *Physics in Medicine and Biology*.

[70] Whiteley JP (2006). An efficient numerical technique for the solution of the monodomain and bidomain equations. *IEEE Transactions on Biomedical Engineering*.

[71] Taggart P, Sutton PM, Opthof T (2000). Inhomogeneous transmural conduction during early ischaemia in patients with coronary artery disease. *Journal of Molecular and Cellular Cardiology*.

[72] James TN, Sherf L (1968). Ultrastructure of the human atrioventricular node. *Circulation*.

[73] Anderson RH, Janse MJ, van Capelle FJ, Billette J, Becker AE, Durrer D (1974). A combined morphological and electrophysiological study of the atrioventricular node of the rabbit heart. *Circulation Research*.

[74] Anderson RH, Ho SY (1998). The architecture of the sinus node, the atrioventricular conduction axis, and the internodal atrial myocardium. *Journal of Cardiovascular Electrophysiology*.

[75] Racker DK (1999). The AV junction region of the heart: a comprehensive study correlating gross anatomy and direct three-dimensional analysis—part I: Architecture and Topography. *The Anatomical Record*.

[76] Anderson RH, Ho SY, Becker AE (2000). Anatomy of the human atrioventricular junctions revisited. *The Anatomical Record*.

[77] Ko YS, Yeh HI, Ko YL (2004). Three-dimensional reconstruction of the rabbit atrioventricular conduction axis by combining histological, desmin, and connexin mapping data. *Circulation*.

[78] Li J, Greener ID, Inada S (2008). Computer three-dimensional reconstruction of the atrioventricular node. *Circulation Research*.

[79] Hucker WJ, McCain ML, Laughner JI, Iaizzo PA, Efimov IR (2008). Connexin 43 expression delineates two discrete pathways in the human atrioventricular junction. *Anatomical Record*.

[80] Lemery R, Guiraudon G, Veinot JP (2003). Anatomic description of Bachmann’s Bundle and its relation to the atrial septum. *American Journal of Cardiology*.

[81] Mitrofanova L, Ivanov V, Platonov PG (2005). Anatomy of the inferior interatrial route in humans. *Europace*.

[82] Sakamoto SI, Nitta T, Ishii Y, Miyagi Y, Ohmori H, Shimizu K (2005). Interatrial electrical connections: the precise location and preferential conduction. *Journal of Cardiovascular Electrophysiology*.

[83] Massing GK, James TN (1976). Anatomical configuration of the His bundle and bundle branches in the human heart. *Circulation*.

[84] Miquerol L, Meysen S, Mangoni M (2004). Architectural and functional asymmetry of the His-Purkinje system of the murine heart. *Cardiovascular Research*.

[85] Tranum-Jensen J, Wilde AA, Vermeulen JT, Janse MJ (1991). Morphology of electrophysiologically identified junctions between purkinje fibers and ventricular muscle in rabbit and pig hearts. *Circulation Research*.

[86] Ansari A, Ho SY, Anderson RH (1999). Distribution of the Purkinje fibres in the sheep heart. *Anatomical Record*.

[87] Tanaka H, Hamamoto T, Takamatsu T (2005). Toward an integrated understanding of the Purkinje fibers in the heart: the functional and morphological interconnection between the Purkinje fibers and ventricular muscle. *Acta Histochemica et Cytochemica*.

[88] Ryu S, Yamamoto S, Andersen CR, Nakazawa K, Miyake F, James TN (2009). Intramural Purkinje cell network of sheep ventricles as the terminal pathway of conduction system. *Anatomical Record*.

[89] Helm P, Beg MF, Miller MI, Winslow RL (2005). Measuring and mapping cardiac fiber and laminar architecture using diffusion tensor MR imaging. *Annals of the New York Academy of Sciences*.

[90] Ramanathan C, Jia P, Ghanem R, Ryu K, Rudy Y (2006). Activation and repolarization of the normal human heart under complete physiological conditions. *Proceedings of the National Academy of Sciences of the United States of America*.

[91] Geerts L, Bovendeerd P, Nicolay K, Arts T (2002). Characterization of the normal cardiac myofiber field in goat measured with MR-diffusion tensor imaging. *American Journal of Physiology—Heart and Circulatory Physiology*.

[92] Jiang Y, Pandya K, Smithies O, Hsu EW (2004). Three-dimensional diffusion tensor microscopy of fixed mouse hearts. *Magnetic Resonance in Medicine*.

[93] Harrington KB, Rodriguez F, Cheng A (2005). Direct measurement of transmural laminar architecture in the anterolateral wall of the ovine left ventricle: new implications for wall thickening mechanics. *American Journal of Physiology—Heart and Circulatory Physiology*.

[94] Anderson RH, Smerup M, Sanchez-Quintana D, Loukas M, Lunkenheimer PP (2009). The three-dimensional arrangement of the myocytes in the ventricular walls. *Clinical Anatomy*.

[95] Jouk PS, Usson Y, Michalowicz G, Grossi L (2000). Three-dimensional cartography of the pattern of the myofibres in the second trimester fetal human heart. *Anatomy and Embryology*.

[96] Wang K, Ho SY, Gibson DG, Anderson RH (1995). Architecture of atrial musculature in humans. *British Heart Journal*.

[97] Ho SY, Anderson RH, Sánchez-Quintana D (2002). Atrial structure and fibres: morphologic bases of atrial conduction. *Cardiovascular Research*.

[98] Sun H, Khoury DS (2001). Electrical conduits within the inferior atrial region exhibit preferential roles in interatrial activation. *Journal of Electrocardiology*.

[99] Fedorov VV, Glukhov AV, Chang R (2010). Optical mapping of the isolated coronary-perfused human sinus node. *Journal of the American College of Cardiology*.

[100] Moe GK, Preston JB, Burlington H (1956). Physiologic evidence for a dual A-V transmission system. *Circulation Research*.

[101] Mazgalev TN, Ho SY, Anderson RH (2001). Anatomic-electrophysiological correlations concerning the pathways for atrioventricular conduction. *Circulation*.

[102] Hucker WJ, Sharma V, Nikolski VP, Efimov IR (2007). Atrioventricular conduction with and without AV nodal delay: two pathways to the bundle of His in the rabbit heart. *American Journal of Physiology—Heart and Circulatory Physiology*.

[103] Simelius K, Nenonen J, Horáček M (2001). Modeling cardiac ventricular activation. *International Journal of Bioelectromagnetism*.

[104] Vigmond EJ, Clements C (2007). Construction of a computer model to investigate sawtooth effects in the Purkinje system. *IEEE Transactions on Biomedical Engineering*.

[105] Sebastian R, Zimmerman V, Romero D (2011). Construction of a computational anatomical model of the peripheral cardiac conduction system. *IEEE Transactions on Biomedical Engineering*.

[106] Bordas R, Gillow K, Lou Q (2011). Rabbit-specific ventricular model of cardiac electrophysiological function including specialized conduction system. *Progress in Biophysics and Molecular Biology*.

[107] Tawara S (1906). *Das Reizleitungssystem Des Saugetierherzens. Eine Anatomisch-Histologische Studie Uber Das Atrioventrikularbundel Und Die Purkinjeschen Faden*.

[108] Spach MS, Huang S, Amstrong SI, Canent RV (1963). Demonstration of peripheral conduction system in human hearts. *Circulation*.

[109] Myerburg RJ, Gelband H, Nilsson K (1972). Physiology of canine intraventricular conduction and endocardial excitation. *Circulation Research*.

[110] Atkinson A, Inada S, Li J (2011). Anatomical and molecular mapping of the left and right ventricular His-Purkinje conduction networks. *The Journal of Molecular and Cellular Cardiology*.

[111] Lemery R, Soucie L, Martin B, Tang ASL, Green M, Healey J (2004). Human study of biatrial electrical coupling: determinants of endocardial septal activation and conduction over interatrial connections. *Circulation*.

[112] Hansson A, Holm M, Blomström P (1998). Right atrial free wall conduction velocity and degree of anisotropy in patients with stable sinus rhythm studied during open heart surgery. *European Heart Journal*.

[113] Boineau JP, Canavan TE, Schuessler RB, Cain ME, Corr PB, Cox JL (1988). Demonstration of a widely distributed atrial pacemaker complex in the human heart. *Circulation*.

[114] Dolber PC, Spach MS (1989). Structure of canine Bachmann’s bundle related to propagation of excitation. *American Journal of Physiology—Heart and Circulatory Physiology*.

[115] De PR, Ho SY, Salerno-Uriarte JA, Tritto M, Spadacini G (2002). Electroanatomic analysis of sinus impulse propagation in normal human atria. *Journal of Cardiovascular Electrophysiology*.

[116] Tapanainen JM, Jurkko R, Holmqvist F (2009). Interatrial right-to-left conduction in patients with paroxysmal atrial fibrillation. *Journal of Interventional Cardiac Electrophysiology*.

[117] Li YG, Grönefeld G, Israel C, Bogun F, Hohnloser SH (2002). Bundle branch reentrant tachycardia in patients with apparent normal His-Purkinje conduction: the role of functional conduction impairment. *Journal of Cardiovascular Electrophysiology*.

[118] Romero D, Sebastian R, Bijnens BH (2010). Effects of the purkinje system and cardiac geometry on biventricular pacing: a model study. *Annals of Biomedical Engineering*.

[119] Lambiase PD, Rinaldi A, Hauck J (2004). Non-contact left ventricular endocardial mapping in cardiac resynchronisation therapy. *Heart*.

[120] van Dam PM, Oostendorp TF, Linnenbank AC, van Oosterom A (2009). Non-invasive imaging of cardiac activation and recovery. *Annals of Biomedical Engineering*.

